# Identification of phlebotomine sand fly blood meals by real-time PCR

**DOI:** 10.1186/s13071-015-0840-3

**Published:** 2015-04-16

**Authors:** Kamila Gaudêncio da Silva Sales, Pietra Lemos Costa, Rayana Carla Silva de Morais, Domenico Otranto, Sinval Pinto Brandão-Filho, Milena de Paiva Cavalcanti, Filipe Dantas-Torres

**Affiliations:** Department of Immunology, Centro de Pesquisas Aggeu Magalhães, Fundação Oswaldo Cruz, Recife, Pernambuco 50740-465 Brazil; Department of Veterinary Medicine, Università degli Studi di Bari, Valenzano, Bari 70010 Italy

**Keywords:** Phlebotomine sand flies, Blood meal, Brazil, Real time PCR

## Abstract

**Background:**

Phlebotomine sand flies are blood-feeding insects of great medical and veterinary significance acting as vectors of *Leishmania* parasites. Studying the blood-feeding pattern of these insects may help in the understanding of their interactions with potential reservoir hosts of *Leishmania* parasites. In this study, we developed real time PCR assays for the identification of sand fly blood meal.

**Methods:**

Six pairs of primers were designed based on *cytochrome b* gene sequences available in GenBank of the following potential hosts: dog, cat, horse, chicken, black rat, and human. Firstly, SYBR Green-based real time PCR assays were conducted using a standard curve with eight different concentrations (i.e., 10 ng, 1 ng, 100 pg, 10 pg, 1 pg, 100 fg, 10 fg and 1 fg per 2 μl) of DNA samples extracted from EDTA blood samples from each target animal. Then, DNA samples extracted from field-collected engorged female sand flies belonging to three species (i.e., *Lutzomyia longipalpis*, *L. migonei* and *L. lenti*) were tested by the protocols standardized herein. Additionally, female sand flies were experimentally fed on a black rat (*Rattus rattus*) and used for evaluating the time course of the detection of the protocol targeting this species.

**Results:**

The protocols performed well with detection limits of 10 pg to 100 fg. Field-collected female sand flies were fed on blood from humans (73%), chickens (23%), dogs (22%), horses (15%), black rats (11%) and cats (2%). Interestingly, 76.1% of the *L. longipalpis* females were positive for human blood. In total, 48% of the tested females were fed on single sources, 31% on two and 12% on three. The analysis of the time course showed that the real time PCR protocol targeting the black rat DNA was able to detect small amounts of the host DNA up to 5 days after the blood meal.

**Conclusions:**

The real time PCR assays standardized herein successfully detected small amounts of host DNA in female sand flies fed on different vertebrate species and, specifically for the black rats, up to 5 days after the blood meal. These assays represent promising tools for the identification of blood meal in field-collected female sand flies.

## Background

Phlebotomine sand flies are blood-feeding insects of great medical and veterinary significance, due to their ability to transmit bacteria, virus, and protozoa to humans and animals [1]. Indeed, besides water and carbohydrates for flight and general metabolism, female sand flies almost always need to take a blood meal for the protein supplementation for egg production [2]. While the preference of certain species of sand flies for a given animal species or group (e.g., mammals or birds) may vary widely, the selective behaviour of some species has been acknowledged. For instance, in a study conducted in Belém (Pará, Brazil), *Lutzomyia flaviscutellata* was the predominant species reported to bite rodents [3]. Nonetheless, most sand fly species for which information is available appear to be generalists rather than specialists in their host range [4-8]. This is the case of *Lutzomyia longipalpis*, the most important vector of *Leishmania infantum* in the Americas [9], making the host choice a matter of availability rather than preference. Understanding the blood-feeding patterns of these insects is of great ecological and epidemiological relevance, as it may provide date on host use and on potential reservoirs of *Leishmania* spp. [10].

Different methods have been traditionally applied to study the blood-feeding behaviour of sand flies, including the precipitin test [4,7,11-13] and ELISA [14-16]. However, these methods present some technical limitations (e.g., the possibility of cross-reactivity between species, the need for producing specific antibodies to several species, and the inability to discover unpredicted hosts) ([10]; and references cited therein).

In light of these limitations, molecular methods have been developed for arthropod blood meal identification, including DNA sequencing, group-specific polymerase chain reaction primers, restriction fragment length polymorphism, real-time polymerase chain reaction, heteroduplex analysis, reverse line-blot hybridization and DNA profiling (reviewed in [17]). Incidentally, several genetic markers have been employed including mitochondrial genes (e.g., cytochrome *b* and cytochrome *c* oxidase subunit I genes), ribosomal RNA genes (e.g., 12S and 16S rDNA) and a nuclear gene (e.g., *prepronociceptin* gene) [17].

In the present study, we developed six uniplex SYBR Green-based real time PCR assays for sand fly blood meal identification using cytochrome *b* as a genetic target. These assays enable the detection of small quantities of the host DNA and represent new tools for the study of vector-host interactions.

## Methods

### Blood samples and sand flies

Blood samples from potential sand fly blood sources (i.e., dog, cat, horse, chicken, black rat, and human) were used as standard DNA. Moreover, a group of female sand flies were experimentally fed on a black rat (*Rattus rattus*) and used for evaluating the detection capacity of the assay targeting this species in function of time (see below).

Out of 24,226 sand flies collected in the framework of a previous study [18], 100 engorged females (92 *L. longipalpis*, seven *Lutzomyia migonei* and one *Lutzomyia lenti*) were used as field samples. In brief, these females were collected from August 2009 to August 2010, using standard CDC light traps in chicken coops, corrals and other animal sheds near human houses in the municipality of Passira (07°59′42″ S, 35°34′51″ O), Pernambuco, northeastern Brazil [18]. In the laboratory, these females were dissected and both head and the last three abdominal segments were used for species identification [19]. The thorax and remaining part of the abdomen of each female were transferred to a 1.5 ml tube and stored at -20°C for molecular processing.

### DNA extraction and quality assessment

Genomic DNA was extracted from female sand flies and animal blood using DNeasy Blood & Tissue kit and QIAamp DNA Blood Mini Kit (Qiagen), respectively. Purified DNA samples were eluted in 100 μl of Tris-EDTA buffer and frozen at -80°C. The quantity and degree of purity of the DNA samples was assessed using a Nanodrop 2000c spectrophotometer (Thermo Scientific).

### Primer designing and real time PCR conditions

Primers targeting each host species (i.e., dog, cat, horse, chicken, black rat, and human) were designed based on cytochrome *b* gene sequences available in GenBank (Table 1), using Primer BLAST (http://www.ncbi.nlm.nih.gov/tools/primer-blast), considering the following criteria: expected PCR product size (70–120 base pairs) and primer melting temperatures (57–63°C).

**Table 1:** **Primers targeting host** ***cytochrome b*** **gene**

**Host species**	**Primers**	**CG content**	**Tm (°C)**	**Product size (bp)**
*Canis lupus familiaris*	f5′ - AGCGCCGTCTAACATCTCTG - 3′	55.45	55.90	118
	r5′ - TGTGGCTGTGTCCGATGTAT - 3′	50.89	59.10	
*Equus caballus*	f5′ - CAGCCAGTGGAACACCCATA- 3′	55.00	59.67	103
	r5′ - TGTTTTCGATGGTGCTTGCG - 3′	50.00	60.04	
*Felis catus*	f5′ - AGAATGGATCTGAGGGGGCT - 3′	55.00	60.03	108
	r5′ - AGGTGTACTGCTGCTAAGGC - 3′	55.00	59.75	
*Gallus gallus*	f5′ - CAGCAGACACATCCCTAGCC - 3′	60.00	60.18	104
	r5′ - GAAGAATGAGGCGCCGTTTG - 3′	55.00	60.18	
*Homo sapiens*	f5′ - AGGCGTCCTTGCCCTATTAC- 3′	55.00	59.53	104
	r5′ - GTGATTGGCTTAGTGGGCG - 3′	55.00	60.39	
*Rattus rattus*	f5′ - GAATTGGGGGCCAACCAGTA - 3′	55.00	59.00	109
	r5′ - TCAATGATTCCGGAGATTGGT - 3′	42.86	57.00	

CG content: guanine-cytosine content; Tm: melting temperature.

DNA-free water was used as no template control (NTC) and unengorged females as negative control. The uniplex real time PCR reactions were run in an ABI PRISM 7000® (Applied Biosystems) and the results analyzed using 7500 software v2.3 (Applied Biosystems). Initially, each reaction consisted of a final volume of 50 μl containing 21 μl of type 1 water, 1 μl of each primer (5 pmol), 25 μl of SYBR Green-PCR Master Mix (Applied Biosystems) and 2 μl of DNA template. Some reactions were also done in a final volume of 25 μl (i.e., 8.5 μl of type 1 water, 1 μl of each primer (5 pmol), 12.5 μl of SYBR Green-PCR Master Mix and 2 μl of DNA template), with no differences in terms of efficiency (data not shown). PCR conditions were: initial denaturation at 95°C for 10 min, then 40 cycles at 95°C for 15 s and at 60°C for 1 min. All samples were tested in duplicate. The cutoff point was defined as the Ct value that corresponds to the defined lower limit of detection of the assay and, any Ct value above this limit, was considered negative.

### Efficiency, specificity, detection limit and time course detection

The amplification efficiency (ε) was calculated using the equation: *ε* = 10^(‐ 1/slope)^ − 1 [20]. In the same way, the specificity (σ) of the assays was determined by using primers for one animal species (target) and DNA from another animal species (templates), being calculated using the equation: *σ* = (1 + *ε*)^ΔCt^; where ∆Ct is the difference in the Ct values of the defined target and the templates. Melt curve was also considered in the specificity analysis. The detection limit of the assays was assessed using 10-fold serial dilutions (10 ng, 1 ng, 100 pg, 10 pg, 1 pg, 100 fg, 10 fg, and 1 fg per 2 μl) of genomic DNA from each animal species. Finally, time course experiments were carried out to determine how long the host DNA could remain detectable by real time PCR. In particular, sand flies (*n* = 50) fed on a black rat were kept in the laboratory for different periods of time (i.e., 1 h, 24 h, 48 h, 72 h, 96 h, 120 h, and 142 h) after the blood meal ingestion. At each time point five female sand flies were taken and subjected to DNA extraction or kept at-20°C until processing.

### DNA sequencing and analysis

PCR products were purified using Pure Link PCR Purification (Invitrogen), sequenced using a Big Dye Terminator v3.1 Cycle Sequencing and analyzed ABI 3100 Genetic Analyzer (Applied Biosystems). Sequences generated were compared with known sequences available in the National Center for Biotechnology Information GenBank by using the Basic Local Alignment Search Tool algorithm (http://www.ncbi.nlm.nih.gov/BLAST).

### Ethical considerations

All the procedures adopted in this study were approved by the human and animal ethics committees of the Centro de Pesquisas Aggeu Magalhães, Oswaldo Cruz Foundation, Recife, Pernambuco, Brazil (CPqAM: CEP 14/13 and CEUA 55/2013).

## Results

**Figure 1:**
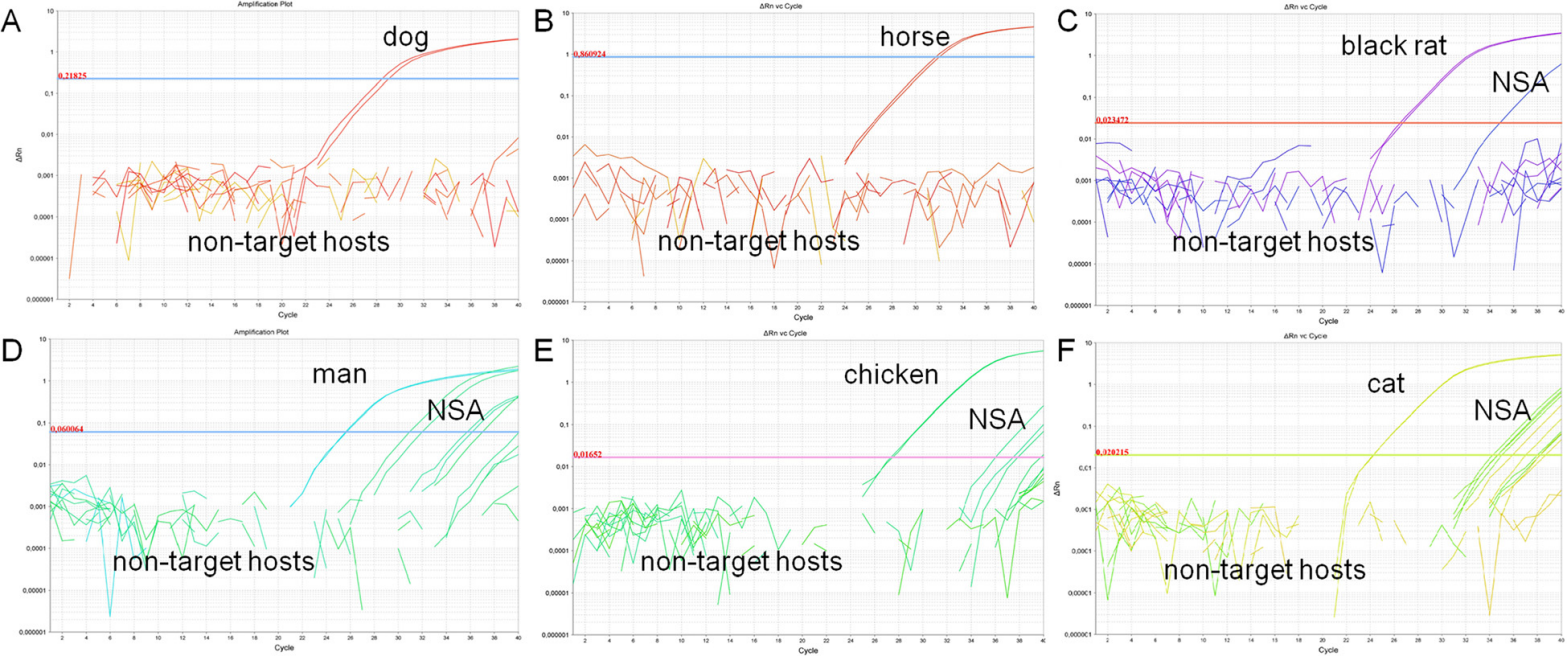
Specificity of the protocols. Specificity assays for each real time PCR protocol targeting different animal species: **A**, dog; **B**, horse; **C**, black rat; **D**, man; **E**, chicken; **F**, cat. Non-specific amplifications (NSA) were determined by melt curve analysis and/or cutoff *Ct* value (for more details, see text).

Each real time PCR protocol amplified successfully the DNA of the target animal species (Figure 1). No amplification was obtained with NTC or with negative controls (unengorged females). Non-specific amplification occurred in some cases: the primers targeting chickens amplified human (Ct 36.2–37.9) and black rat (Ct 37.6); the primers targeting cats amplified horse (Ct 37.2–38.6), dog (Ct 35.9–38.2), black rat (Ct 38.0–38.2), chicken (Ct 34.8–35.2) and human (Ct 34.4–34.7) DNA; and the primers targeting humans amplified dog (37.1), cat (Ct 31.0–32.0), and black rat (Ct 35.7–36.1). However, the non-specific amplifications above occurred usually in later cycles and could be distinguished by the Ct value (above defined lower limit of detection of each corresponding assay) and/or by melt curve analysis (as compared to the standard). Moreover, the specificity of the assays was also confirmed by DNA sequence analysis, which showed high levels of sequence identity (96–100%) with corresponding sequences available in GenBank (accession number: KJ185407.1, KF282339.1, KF964328.1, AB194817.1, KF038166.1, and KJ522809.1).

**Figure 2:**
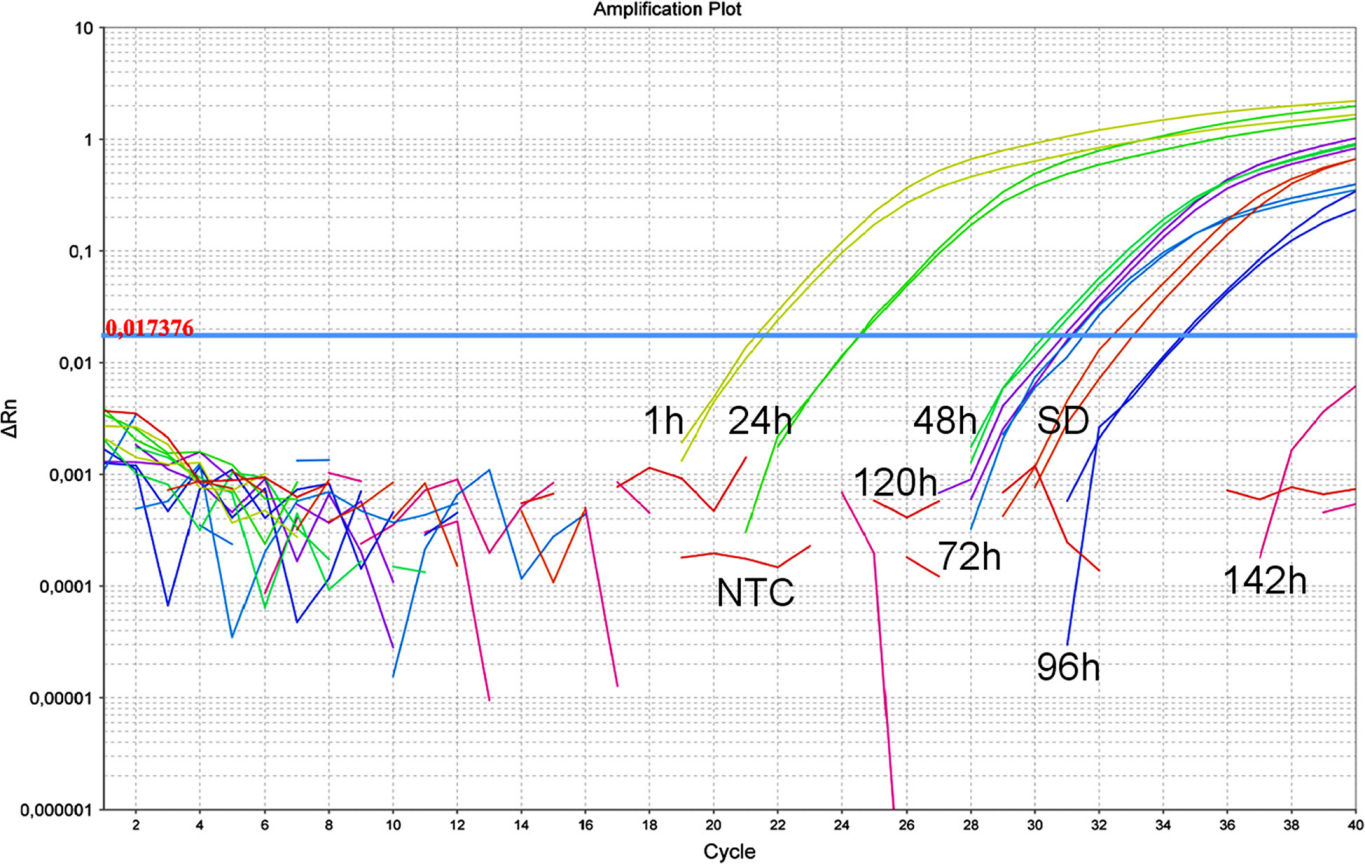
Time course of the detection. Time course of the detection (from 1 h to 142 h) of black rat DNA in experimentally fed sand flies. NTC, no template control. SD, standard DNA (1 pg, black rat DNA extracted from blood).

The detection limit of the assays ranged from 10 pg to 100 fg, with an acceptable level of homogeneity among replicates and reaction efficiency (Table 2). Remarkably, time course experiments showed that the real time PCR assay targeting black rat DNA was capable of detecting small amounts of the host DNA (~1 pg) up to 120 h after the blood feeding (Figure 2).

**Table 2:** **Efficiency and detection limit**

**Target host**	**Slope**	**R** ^**2**^	ε **(%)**	**Detection limit (per 2 μl)**
Dog	-3.58 ± 0.69	0.93 ± 0.10	96.3 ± 30.8	1 pg
Horse	-3.88 ± 0.61	0.96 ± 0.02	83.28 ± 14.74	10 pg
Cat	-3.37 ± 0.16	0.93 ± 0.02	96.89 ± 4.13	1 pg
Black rat	-4.29 ± 0.16	0.94 ± 0.04	71.06 ± 3.32	1 pg
Chicken	-3.65 ± 0.65	0.96 ± 0.06	92.61 ± 27.24	10 pg
Man	-3.84 ± 0.53	0.83 ± 0.23	84.17 ± 15.74	100 fg

Slope, R^2^, amplification efficiency, and detection limit of the assays.

Among field-collected engorged female sand flies, 91% were positive and 9% negative for any host DNA. In particular, 73% of them were fed on humans, 23% on chickens, 22% on dogs, 15% on horses, 11% on black rats and 2% on cats. Interestingly, most *L. longipalpis* females were fed on humans (76.1%), followed by chickens (19.6%), dogs (16.3%), horses (16.3%), black rats (12%) and cats (2.2%).

In total, 48% of the field-collected female sand flies were positive for one animal species, 31% for two and 12% for three. Among females that were fed on one source, the majority contained human blood (73%), followed by chicken (10.4%) and dog blood (6.2%). Among those fed on two sources, most of them were positive for human + horse (7), human + chicken (7), human + dog (8) and human + rat (4). Finally, most females fed on three sources were positive for human + dog + chicken (5), followed by human + horse + rat (3), human + dog + cat (1), human + horse + chicken (1), and human + chicken + rat (1).

## Discussion

In the present study we developed SYBR Green-based real time PCR assays for the identification of female sand fly blood meals. Remarkably, the assays were capable of detecting small amounts of host DNA in field-collected engorged females stored at -20°C for ~4 years. The good performance of the assays developed herein allowed the detection of as little as 100 fg per reaction mixture (i.e., 2 μl) of the host DNA (i.e., human). Indeed, the detection limit of our assays is fairly equivalent to that reported by some authors (10 pg in Ref. [10]; 1 pg in Ref. [21]), confirming the usefulness of the cytochrome *b* gene as a genetic target for blood meal identification in sand flies, as previously demonstrated with conventional PCR protocols [21-24].

Several methodologies have been used to detect the blood meal in arthropod vectors, such as mosquitoes [17], but there are some biological differences that make it difficult to extrapolate the results for sand flies, including the lower amount of blood imbibed by sand flies (1 μl or less) [25] than mosquitoes (2–6 μl) [26]. Additionally, the blood digestion in haematophagous insects may result in DNA denaturation, therefore impairing the detection of the host DNA some days after the blood meal [27,28]. It is thus desirable to have a technique that is sensitive enough to allow the detection of minimal amounts of DNA, even some days after the ingestion of the blood. Indeed, it is difficult to estimate the time elapsed since the last blood meal, particularly in field-collected female sand flies with no visible blood in the abdomen. The results of time course experiments obtained herein demonstrated that the protocol targeting black rats allowed the detection of the host DNA up to 5 days after the blood feeding. This is consistent with other published assays for mosquito and sand fly blood meal identification, which generally were able to detect the host DNA for up to 1–4 days [10,21,22,27-29]. Interestingly, a PCR heteroduplex assay was developed to identify avian derived mosquito blood meals, being capable of detecting the host DNA for up to 7 days [30]. The authors suggested that the greater amount of host DNA in the avian blood meal persists for a longer period than in a mammalian blood meal.

The catholic feeding behavior of *L. longipalpis* is well acknowledged [9]. Accordingly, most females belonging to this species analyzed in the current study were positive for humans, followed by chickens, dogs, horses, black rats and cats. Interestingly enough, most field-collected *L. longipalpis* females were trapped in chicken coops near human houses, being 19.2% of the human blood-positive females also positive for chicken blood. This data may suggest that the establishment of chicken coops near human houses may increase the risk of exposure to sand flies. Certainly, studying the blood feeding behaviour of sand fly vectors may help in understanding host-vector interactions and possibly the transmission dynamics of *Leishmania* parasites [31,32].

## Conclusions

In conclusion, the SYBR Green-based real time PCR assays standardized herein represent promising tools for blood meal identification in field-collected sand flies. Indeed, these assays successfully detected small amounts of host DNA in female sand flies fed on different vertebrate species and, specifically for black rats, up to 5 days after the blood meal. As a perspective, it would be valuable to increase the efficiency of these assays for blood meal quantification purposes. Finally, from a cost-benefit perspective, a multiplex real time PCR assay should be standardized for the simultaneous detection of blood meals from different hosts in sand flies as well in other blood feeding arthropods. In this perspective, the use of TaqMan probes and, perhaps, designing new primers (e.g., for cats and humans) would be more appropriate to increase the specificity of the assay.
